# Saponins with Neuroprotective Effects from the Roots of *Pulsatilla cernua*

**DOI:** 10.3390/molecules17055520

**Published:** 2012-05-09

**Authors:** Jian-Yu Liu, Ying-Li Guan, Li-Bo Zou, Yi-Xia Gong, Hui-Ming Hua, Yong-Nan Xu, Hui Zhang, Zong-Gui Yu, Wen-Hao Fan

**Affiliations:** 1 Key Laboratory of Structure-Based Drug Design & Discovery, Ministry of Education, Shenyang Pharmaceutical University, Shenyang 110016, China; Email: burningice@126.com (J.-Y.L.); 2 School of Pharmaceutical Engineer, Shenyang Pharmaceutical University, Shenyang 110016, China; 3 Department of Pharmarcy and Food Science, Tonghua Normal University, Tonghua 134000, China; 4 Department of Pharmacology, School of Life Science and Biopharmaceutics, Shenyang Pharmaceutical University, Shenyang 110016, China; 5 School of Traditional Chinese Materia Medica, Shenyang Pharmaceutical University, Shenyang 110016, China

**Keywords:** *Pulsatilla cernua*, saponin, neuroprotective effect, Alzheimer’s disease, β-amyloid

## Abstract

Four new oleanene-type triterpenoid saponins together with six known saponins were isolated from the roots of *Pulsatilla cernua* and their structures were elucidated on the basis of spectroscopic data, including 2D NMR spectra and chemical evidence. Among these one of the aglycones (gypsogenin) is reported for the first time from this genus. Some of these compounds showed significant neuroprotective effects against the cytotoxicity induced by β-amyloid_25–35_ (Aβ_25–35_) on human neuroblastoma SH-SY5Y cells.

## 1. Introduction

**Figure 1:**
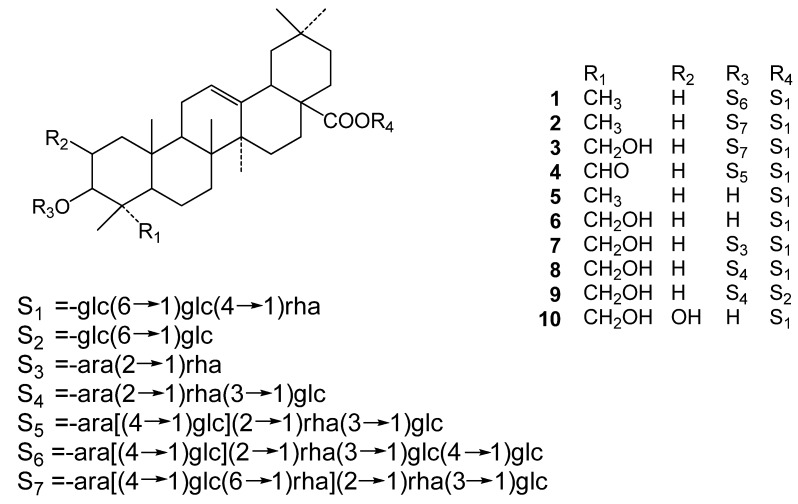
Chemical structures of compounds **1–10**.

*Pulsatilla cernua* (Thunb.) Bercht. et Opiz. (Ranunculaceae) is a traditional medicinal plant in northeastern China and Korea. The roots are used for the treatment of amoebic dysentery, malaria and chills [1]. Previous phytochemical investigations on this plant have reported a number of triterpenoid saponins [1,2,3,4,5,6,7,8,9]. Modern researches have shown that the chemical components of *P. cernua* had strong biological activities, and especially the saponin-enriched fraction with some active components showing remarkable effects in the treatment of Alzheimer’s disease (AD) [10,11]. In this paper, the *n*-BuOH-soluble fraction of this plant was investigated to yield four new saponins, together with six known saponins which were identified as cussonoside B (**5**) [12], pulsatiloside C (**6**) [13], hederacochiside C (**7**) [1,7], patrinia saponin H3 (**8**) [7], aralia saponin 3 (**9**) [14], bayogenin 28-*O-α-*L- rhamnopyranosyl(1→4)-*β*-D-glucopyranosyl(1→6)-*β*-D-glucopyranosyl ester (**10**) [15] (Figure 1). This paper describes the structural determination of the four new saponins and the neuroprotective effect of some of these compounds against the cytotoxicity induced by β-amyloid_25–35_ (Aβ_25–35_) on human neuroblastoma SH-SY5Y cells.

## 2. Results and Discussion

Compounds **1–4** were obtained as white amorphous powders. Acid hydrolysis of these compounds with 1 M HCl gave arabinose, rhamnose and glucose, which were identified by TLC comparison with authentic samples. The *β*-anomeric configurations for the D-glucose, and the *α*-anomeric configurations for L-arabinose were determined by their ^3^*J*_H1,H2_ coupling constants of 7–8 Hz. The *α*-anomeric configuration of L-rhamnose was judged by the chemical shift of C-5 (δ_C_ 69–70) [16]. The absolute configuration of the saccharides was determined to be D- for glucose, L- for rhamnose and arabinose by GC analysis of chiral derivatives in the hydrolysate of these compounds.

**Figure 2:**
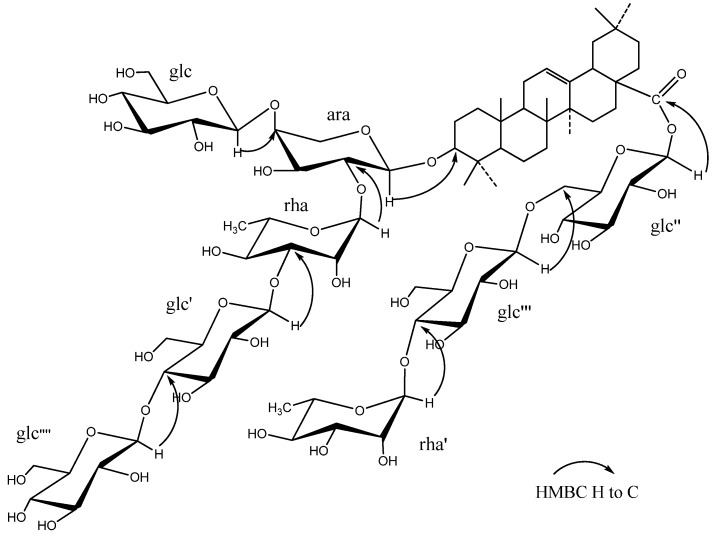
Key HMBC correlations (from H to C) of compound **1**.

The molecular formula of **1** was determined as C_77_H_126_O_40_ from HR-TOF-MS (*m/z* 1689.7773 [M−H]^−^, calc. 1689.7752). The compound displayed 77 carbon signals in the ^13^C-NMR spectrum, of which 30 were assigned to the aglycone and the remaining 47 to the sugar moieties. The seven methyl carbon signals at *δ*_C_ 15.4, 17.0, 17.2, 23.5, 25.8, 28.0 and 32.9, and the two olefinic carbon signals at *δ*_C_ 122.9 and 144.2, coupled with the ^1^H-NMR data, seven tertiary methyl proton singlets at *δ*_H_ 0.82, 0.84, 0.84, 1.03, 1.12, 1.19, and 1.26, and a broad triplet-like olefinic proton signal at *δ*_H_ 5.35 (brs),indicated that the aglycone possessed an olean-12-ene skeleton. C18 configuration was recognized as H18*β* by the chemical shift of C12, C13 and C16 due to the *γ*-gauche interactions [16]. The ^1^H-NMR spectrum showed eight anomeric proton signals at *δ*_H_ 4.61 (1H, d, *J =* 6.0 Hz), 4.96 (1H, d, *J =* 7.2 Hz), 5.10 (1H, d, *J =* 7.8 Hz), 5.15 (1H, d, *J =* 7.8 Hz) 5.42 (1H, d, *J =* 7.8 Hz), 5.84 (1H, brs), 6.19 (1H, brs), 6.22 (1H, d, *J =* 7.8 Hz), as well as two methyl doublets of rhamnose at *δ*_H _1.52 (3H, d, *J =* 6.0 Hz) and 1.66 (3H, d, *J =* 6.0 Hz), and the corresponding anomeric carbon signals at *δ*_C_ 95.4, 101.3, 102.5, 104.6 104.8, 105.1, 106.3, 106.4, respectively (Table 1). The chemical shifts of *δ*_C_ 88.3 (C-3) and 176.5 (C-28) revealed that **1** was a bidesmosidic saponin with a glycosidic linkage at C-3 through an *O*-heterosidic bond and at C-28 through an ester bond. The chemical shift of sugar moieties was comfirmed on the basis of the HSQC-TOCSY correlations. The linkage of the sugar moiety at C-3 of the aglycone was established from the HMBC correlations between *δ* 4.61 (1H, d, *J =* 6.0 Hz, ara H-1) and *δ* 88.4 (C-3), *δ* 5.10 (1H, d, *J =* 7.8 Hz, glc H-1) and *δ* 79.9 (ara-4), *δ*6.19 (1H, brs, rha H-1) and *δ* 75.7 (ara-2), *δ* 5.42 (1H, d, *J =* 7.8 Hz, glc′ H-1) and *δ* 83.0 (rha-3), *δ*5.15 (1H, d, *J =* 7.8 Hz, glc″″ H-1) and *δ* 80.9 (glc′-4), and the linkage at C-28 was established from the HMBC correlations between *δ*6.22 (1H, d, *J =* 7.8 Hz, glc″ H-1) and *δ* 176.5 (C-28), 4.96 (1H, d, *J =* 7.2 Hz, glc″′ H-1) and *δ* 68.9 (glc″-6), 5.84 (1H, brs, rha′ H-1) and *δ* 77.9 (glc″′-4) (Figure 2). Based on the above evidence, the structure of **1** was determined to be oleanolic acid 3-*O-β*-D-glucopyranosyl(1→4)-*β*-D-gluco-pyranosyl(1→3)-*α-*L-rhamnopyranosyl(1→2)[*β-*D-glucopyranosyl(1→4)]-*α-*L*-*arabinopyranosyl-28-*O*- *α-*L-rhamnopyranosyl(1→4)-*β*-D-glucopyranosyl(1→6)-*β*-D-glucopyranosyl ester. 

The molecular formula of **2** was determined as C_77_H_126_O_39_ from HR-TOF-MS (*m/z* 1673.7813 [M−H]^−^, calc. 1673.7803). The NMR spectra of aglycone were in good agreement with those of **1**. The ^1^H-NMR spectrum showed eight anomeric proton signals at *δ*_H_ 4.76 (1H，d, *J =* 6.0 Hz), 4.96 (1H, overlapped), 5.11 (1H, d, *J =* 7.2 Hz), 5.39 (1H, brs), 5.40 (1H, d, *J =* 7.8 Hz), 5.85 (1H, brs), 6.21 (1H，overlapped), 6.23 (1H, brs), as well as three methyl doublets of rhamnose at *δ*_H_ 1.50 (3H, d, *J =* 6.0 Hz), 1.55 (3H, d, *J =* 6.0 Hz), 1.67 (3H, d, *J =* 6.0 Hz), and the corresponding carbon signals at *δ*_C_ 95.4, 101.4, 102.6, 102.7, 104.8, 104.9, 105.2, 106.4, respectively (Table 2). The linkage of the sugar moieties at C-3 and C-28 of the aglycone was established from the HMBC correlations. Based on the above evidence, the structure of **2** was determined to be oleanolic acid 3-*O-β*-D-glucopyranosyl(1→3)-*α-*L-rhamnopyranosyl(1→2)[*α-*L-rhamnopyranosyl(1→6)-*β-*D-glucopyranosyl(1→4)]-*α-*L*-*arabinopyranosyl-28-*O*-*α-*L-rhamnopyranosyl(1→4)-*β*-D-glucopyranosyl(1→6)-*β*-D-glucopyranosyl ester.

The molecular formula of **3** was determined as C_77_H_126_O_40_ from HR-TOF-MS (*m/z* 1689.7736 [M−H]^−^, calc. 1689.7752). The NMR spectra were almost similar to those of **2**, apart from the change of the methyl group (*δ*_H_ 1.27 and *δ*_C_ 28.2) in **2** to hydroxymethyl group (*δ*_H_ 3.89, 4.21 and *δ*_C_ 64.0) in **3**, suggesting the hydrogen at C-23 in **2** was substituted by the hydroxyl group in **3**. Thus, the aglycone of **3** was identified as hederagenin. 

**Table 1:** Spectroscopic data of **1** and **4** (sugar moieties, *δ* in ppm, *J* in Hz) in pyridine-*d*_5_.

	1		4			1		4	
	*δ*_C_	*δ*_H_	*δ*_C_	*δ*_H_		*δ*_C_	*δ*_H_	*δ*_C_	*δ*_H_
C3-					glc″″-1	104.8	5.15(1H,d,7.8)		
ara-1	105.1	4.61(1H,d,6.0)	104.7	4.91(1H,d,6.0)	2	74.5	3.95(1H,m)		
2	75.7	4.15(1H,m)	75.5	4.42(1H,m)	3	78.0	4.20(1H,m)		
3	73.8	4.33(1H,m)	74.1	4.55(1H,m)	4	71.2	4.89(1H,brs)		
4	79.1	4.74(1H,dd,2.4,9.6)	80.7	4.30(1H,m)	5	78.2	4.10(1H,m)		
5	65.1	3.69(1H,d,11), 4.40(1H,m)	66.4	3.62(1H,d,6.0), 4.20(1H,m)	6	62.2	4.27(1H,m), 4.53(1H,m)		
glc-1	106.4	5.10(1H,d,7.8)	105.0	4.99(1H,d,7.8)	C28-				
2	75.3	4.15(1H,m)	74.6	4.02(1H,m)	glc″-1	95.4	6.22(1H,d,7.8)	95.8	6.14(1H,d,7.8)
3	78.2	4.19(1H,m)	78.6	4.20(1H,m)	2	74.3	4.15(1H,m)	74.2	4.15(1H,m)
4	71.0	4.85(1H,m)	71.6	4.20(1H,m)	3	78.5	4.40(1H,m)	78.9	4.40(1H,m)
5	78.5	3.90(1H,m)	78.4	3.90(1H,m)	4	70.6	4.20(1H,m)	71.0	4.33(1H,m)
6	62.3	4.37(1H,m), 4.53(1H,m)	62.5	4.25(1H,m), 4.45(1H,m)	5	77.8	3.90(1H,m)	78.4	3.92(1H,m)
rha-1	101.3	6.19(1H,brs)	101.3	6.13(1H,brs)	6	69.0	4.34(1H,m), 4.63(1H,m)	69.4	4.33(1H,m), 4.82(1H,m)
2	71.4	4.90(1H,m)	72.0	4.65(1H,m)	glc″′-1	104.6	4.96(1H,d,7.2)	103.1	4.90(1H,d,7.8)
3	83.0	4.74(1H,dd,2.4,9.6)	83.8	4.69(1H,dd,6.0,12.0)	2	75.1	3.95(1H,m)	74.9	3.93(1H,m)
4	72.7	4.50(1H,m)	73.2	4.35(1H,m)	3	76.5	4.15(1H,m)	76.7	4.13(1H,m)
5	69.5	4.95(1H,m)	69.6	4.65(1H,m)	4	78.0	4.10(1H,m)	78.2	4.09(1H,m)
6	18.3	1.52(3H,d,6.0)	18.6	1.50(3H,d,6.0)	5	76.9	3.61(1H,d,9.0)	77.3	3.57(1H,d,9.0)
glc′-1	106.3	5.42(1H,d,7.8)	106.7	5.11(1H,d,7.8)	6	61.0	4.05(1H,m), 4.17(1H,m)	61.5	4.05(1H,m), 4.15(1H,m)
2	75.2	3.95(1H,m)	75.8	3.90(1H,m)	rha′-1	102.5	5.84(1H,brs)	102.4	5.76(1H,brs)
3	76.5	4.30(1H,m)	76.7	4.30(1H,m)	2	72.3	4.69(1H,m)	72.7	4.65(1H,m)
4	81.0	4.35(1H,m)	69.7	4.40(1H,m)	3	72.5	4.50(1H,m)	72.9	4.55(1H,m)
5	76.3	4.10(1H,m)	77.0	4.10(1H,m)	4	73.7	4.30(1H,m)	74.1	4.35(1H,m)
6	61.7	3.84(1H,m), 4.43(1H,m)	62.0	3.84(1H,m), 4.43(1H,m)	5	70.1	4.31(1H,m)	70.5	4.93(1H,m)
					6	18.3	1.66(3H,d,6.0)	18.7	1.61(3H,d,6.0)

**Table 2:** Spectroscopic data of **2** and **3** (sugar moieties, *δ* in ppm, *J* in Hz) in pyridine-*d*_5_.

	2		3			2		3	
	*δ*_C_	*δ*_H_	*δ*_C_	*δ*_H_		*δ*_C_	*δ*_H_	*δ*_C_	*δ*_H_
C3-					glc′-1	106.4	5.40(1H,d,7.8)	106.7	5.39(1H, *O*)
ara-1	105.2	4.76(1H,d,6.0)	104.9	4.95(1H,d,6.0)	2	75.4	3.90(1H,m)	75.4	3.90(1H,m)
2	75.2	4.49(1H,m)	75.3	4.49(1H,m)	3	78.2	4.19(1H,m)	78.2	4.20(1H,m)
3	74.0	4.29(1H,m)	74.0	4.25(1H,m)	4	71.7	4.85(1H,m)	71.8	4.25(1H,m)
4	81.9	4.22(1H,m)	82.0	4.19(1H,m)	5	78.2	4.15(1H,m)	78.2	4.15(1H,m)
5	65.8	3.75(1H,d,30), 4.16(1H,m)	66.6	3.60(1H,m), 4.18(1H,m)	6	61.8	4.38(1H,m), 4.49(1H,m)	61.8	4.36(1H,m), 4.42(1H,m)
glc-1	104.9	5.11(1H,d,7.2)	105.1	5.06(1H,d,7.8)	C28-				
2	73.9	4.10(1H,m)	72.6	4.49(1H,m)	glc″-1	95.6	6.21(1H, *O*)	95.7	6.20(1H,d,8.4)
3	78.2	4.19(1H,m)	78.2	4.15(1H,m)	2	74.7	4.15(1H,m)	74.9	4.15(1H,m)
4	71.2	4.40(1H,m)	71.0	4.25(1H,m)	3	78.6	4.39(1H,m)	78.8	4.45(1H,m)
5	77.0	4.00(1H,m)	76.8	4.00(1H,m)	4	70.8	4.25(1H,m)	70.8	4.25(1H,m)
6	68.5	3.91(1H,m), 4.58(1H,m)	68.6	3.90(1H,m), 4.57(1H,d,9.0)	5	78.0	4.00(1H,m)	78.2	3.90(1H,m)
rha″-1	102.7	5.39(1H,brs)	102.8	5.37(1H,brs)	6	69.1	4.32(1H,m), 4.60(1H,m)	69.2	4.29(1H,m), 4.63(1H,m)
2	71.8	3.90(1H,m)	72.0	4.85(1H,m)	glc″′-1	104.8	4.96(1H, *O*)	104.9	4.92(1H, *O*)
3	71.8	4.85(1H,m)	72.0	4.65(1H,m)	2	75.2	3.95(1H,m)	75.3	3.95(1H,m)
4	73.9	4.29(1H,m)	73.1	4.44(1H,m)	3	76.4	4.13(1H,m)	76.5	4.15(1H,m)
5	69.8	4.25(1H,m)	69.9	4.21(1H,m)	4	78.1	4.10(1H,m)	78.2	4.00(1H,m)
6	18.4	1.49(3H,d,6.0)	18.5	1.51(3H,d,6.0)	5	77.1	3.61(1H,d,12)	77.2	3.62(1H,m)
rha-1	101.4	6.23(1H,brs)	101.4	6.23(1H,brs)	6	61.2	4.05(1H,m), 4.17(1H,m)	61.3	4.05(1H,m), 4.17(1H,m)
2	71.6	4.25(1H,m)	71.7	3.90(1H,m)	rha′-1	102.7	5.85(1H,brs)	102.9	5.81(1H,brs)
3	83.5	4.70(1H,m)	83.5	4.75(1H,dd,3.0,9.6)	2	72.5	4.63(1H,m)	72.6	4.65(1H,m)
4	73.0	4.43(1H,m)	72.8	4.17(1H,m)	3	72.7	4.49(1H,m)	72.8	4.50(1H,m)
5	69.6	4.59(1H,m)	69.7	4.06(1H,m)	4	73.8	4.30(1H,m)	73.9	4.28(1H,m)
6	18.5	1.54(3H,d,6.0)	18.7	1.55(3H,d,6.0)	5	70.2	4.90(1H,m)	70.4	4.91(1H,m)
					6	18.4	1.67(3H,d,6.0)	18.6	1.66(3H,d,6.0)

The NMR spectra of sugar moieties were in good agreement with those of **2** (Table 2). The linkage of the sugar moieties at C-3 and C-28 of the aglycone was also established from the HMBC correlations, and the structure of **3** was determined to be hederagenin 3-*O-β*-D-glucopyranosyl(1→3)-*α-*L-rhamnopyranosyl(1→2)[*α-*L-rhamnopyranosyl(1→2)-*β-*D-glucopyranosyl(1→4)]-*α-*L*-*arabinopyranosyl-28-*O*-*α-*L-rhamnopyranosyl(1→4)-*β*-D-glucopyranosyl(1→6)-*β*-D-glucopyranosyl ester.

The molecular formula of **4** was determined as C_71_H_114_O_36_ from HR-TOF-MS (*m/z* 1541.6973 [M−H]^−^, calc. 1541.7017). The NMR spectra of aglycone were also similar to those of **2**, apart from the change of the methyl group (*δ*_H_ 1.27 and *δ*_C_ 28.2) in **2** to aldehyde group (*δ*_H_ 9.67 and *δ*_C_ 205.5) in **4**. Thus, the aglycone of **4** was identified as gypsogenin. Compared with **2**, the NMR spectra of sugar moieties were almost similar, only except for the absent of the terminal rhamnose on the sugar chain linked to C-3 of aglycone (Table 1). Thus, the structure of **4** was determined to be gypsogenin 3-*O-β*-D-glucopyranosyl(1→3)-*α-*L-rhamnopyranosyl(1→2)[*β-*D-glucopyranosyl(1→4)]-*α-*L*-*arabinopyranosyl-28-*O*-*α-*L-rhamnopyranosyl(1→4)-*β*-D-glucopyranosyl(1→6)-*β*-D-glucopyranosyl ester.

One of the major pathological features of AD is the appearance of senile plaques characterized by extracellular aggregation of Aβ fibrils, 39 to 43 amino acid peptides derived from the amyloid precursor protein (APP). It has been shown that Aβ_25–35_, a peptide comprising 11 residues within Aβ_1–42_, aggregates and retains the neurotoxin activities just like the full-length Aβ. Therefore Aβ_25–35_ was used in the experiment of this study [17,18]. Human neuroblastoma SH-SY5Y cell is a widely and extensively used target cell line in the assessment of neurotoxicity and neuroprotection.

**Figure 3:**
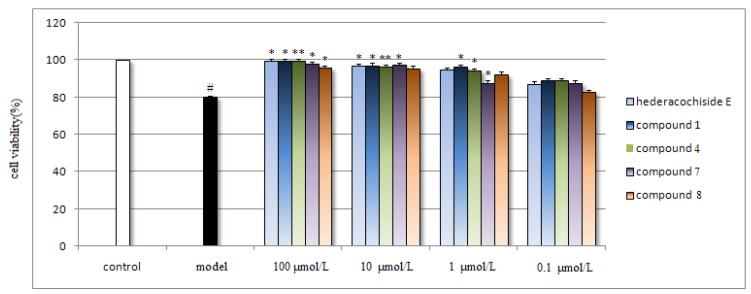
Effect of compounds **1**, **4**, **7** and **8** on cell ciability in Aβ_25–35_-induced cytotoxicity.

Neuroprotective effects of compounds against the cytotoxicity induced by Aβ_25–35_ on SH-SY5Y cell were tested by the MTT assay. Due to its knwon significant neuroprotective effects against Aβ [11], hederacochiside E was used as positive control. In the Aβ model group, the cell viability were decreased by ~20% compared with the control group. While in contrast, the cell viability of the groups treated by saponin **1**, **4**, **7** and **8** were increased by ~20% compared with the model group (*p* < 0.05, *p* < 0.01, *p* < 0.05, *p* < 0.05, respectively) at the highest concentration (100 μmol/L). In addition, the results of **1** and **4** were similar with the compound hederacochiside E (Figure 3). While other compounds showed no effects (data not shown). The results indicated that **1**, **4**, **7** and **8** showed significant effect against the cytotoxicity induced by Aβ_25–35_ on SH-SY5Y cell and can be further investigated. 

Compounds **1**, **4**, **7** and **8** reduced Aβ_25–35_-induced cytotoxicity in human SH-SY5Y cells. The cultured cells were pre-incubated with different compounds for 18 h, and exposed to 20 μM Aβ_25–35_ for an additional 30 h, then 15 μL of MTT stock solution was added to the culture medium for 4 h at 37 °C. Cell viability was determined by measuring MTT reduction. Results are shown as the mean ± SEM and represent six independent experiments. *p < 0.05, **p < 0.01 *vs.* model group (treated with Aβ_25–35_); # *p* < 0.05 *vs.* control group.

## 3. Experimental

### 3.1. General

The NMR spectra were measured in pyridine-*d*_5_, on a Bruker AV600 instrument. ESI-MS spectra were recorded on Waters Quattro micro API LC/MS/MS spectrometer (Waters, USA). HR-TOF-MS spectra were performed on Agilent LC/MS spectrometer (Agilent, USA). HPLC was performed on JAI LC9103 Recycling preparative HPLC (Japan Analytical Industries, Japan) equipped with JAIGEL-ODS-AP-P column and JAIGEL-GS310 column using a JAI refractive index detector and a JAI UV-3702 detector with MultiChro 2000 workstation. TLC was performed on pre-coated GF_254_ plates (Merck, Germany) and detected by spraying with 10% H_2_SO_4_ followed heating. GC analyses were performed using an Agilent GC 6890 instrument on an HP-5 column (320 μm × 30 m, 0.25 μm). 

### 3.2. Plant Material

The roots of *P. cernua* were collected in May 2007 at Qingyuan, Fushun, Liaoning, China, and authenticated by Professor Jin-Cai Lu (The School of Traditional Chinese Materia Medica, Shenyang Pharmaceutical University). A voucher specimen has been deposited in our laboratory (voucher No. pc-2007-001).

### 3.3. Extraction and Isolation

The air-dried and pulverized roots of *P. cernua* (5 kg) were extracted three times at 80 °C with 50% aqueous EtOH (20 L and 4 h each time), and then the extracts were combined and concentrated under reduced pressure at 60 °C *in vacuo* to give a residue (550 g), which was then suspended in water, and partitioned successively with petroleum ether, CH_2_Cl_2_, EtOAc and *n*-BuOH in the same volume (5 L) three times, respectively. The *n*-BuOH-soluble fraction (160.5 g) was subjected to column chromatography on D101 macroporous resin, and eluted with a gradient of aqueous EtOH (30%, 50%, 70%, 95% EtOH, respectively) to give four fractions. The 50% eluting fraction was subjected to silica gel chromatography, eluting with a gradient of CHCl_3_-MeOH-H_2_O (100:10:1→100:60:10) to give three fractions (Fr.1 to Fr.3), and the 70% eluting fraction was likewise treated with a gradient of CHCl_3_-MeOH-H_2_O (100:10:1→100:30:4) to give one fraction (Fr.4). Fr.3 was then subjected to HPLC with MeOH-CH_3_CN-H_2_O (3:2:3) as eluent and each subfraction was further purified on recycling preparative HPLC with MeOH-CH_3_CN (3:2) to yield **1** (60 mg), **3** (5.6 mg), **4** (8.6 mg), **5** (15 mg), **6** (36 mg), **7** (27 mg), **8** (18 mg), **9** (10 mg) and **10** (5 mg). Fr.4 was then subjected to HPLC with MeOH-CH_3_CN-H_2_O (3:2:1) and further purified on recycling preparative HPLC with MeOH-CH_3_CN (3:2) to yield **2** (6 mg).

### 3.4. Spectral Data

*Oleanolic acid 3-O-β-D-glucopyranosyl(1→4)-β-D-glucopyranosyl(1→3)-α-L-rhamnopyranosyl(1→2)[β-D-glucopyranosyl(1→4)]-α-L-arabinopyranosyl-28-O-α-L-rhamnopyranosyl(1→4)-β-D-glucopyranosyl(1→6)-β-D-glucopyranosyl ester* (**1**). White amorphous powder; mp. 270–273 °C; HR-TOF-MS *m/z* 1689.7773 [M−H]^−^ (calc. for C_77_H_126_O_40_, *m/z* 1689.7752); ^1^H-NMR of aglycone δ: 0.89 (1H, m, H-1a), 1.45 (1H, m, H-1b), 1.85 (1H, m, H-2a), 2.05 (1H, m, H-2b), 3.19 (1H, dd, *J* = 3.6, 11.0 Hz, H-3), 0.72 (1H, d, *J* = 12.0 Hz, H-5), 1.60 (2H, m, H-6), 1.29 (1H, m, H-7a), 1.40 (1H, m, H-7b), 1.61 (1H, m, H-9), 0.88 (2H, m, H-11), 5.35 (1H, s, H-12), 1.16 (1H, m, H-15a), 2.27 (1H, m, H-15b), 1.90 (1H, m, H-16a), 2.05 (1H, m, H-16b), 3.13 (1H, d, *J* = 12.0 Hz, H-18), 1.21 (1H, m, H-19a), 1.73 (1H, m, H-19b), 0.90 (1H, m, H-21a), 1.09 (1H, m, H-21b), 1.75 (1H, m, H-22a), 1.85 (1H, m, H-22b), 1.26 (3H, s, H-23), 1.12 (3H, s, H-24), 0.84 (3H, s, H-25), 1.03 (3H, s, H-26), 1.20 (3H, s, H-27), 0.84 (3H, s, H-29), 0.82 (3H, s, H-30); ^13^C-NMR of aglycone *δ*: 38.5 (C-1), 26.5 (C-2), 88.3 (C-3), 39.3 (C-4), 55.8 (C-5), 18.3 (C-6), 32.9 (C-7), 39.7 (C-8), 47.8 (C-9), 36.8 (C-10), 23.6 (C-11), 122.6 (C-12), 144.9 (C-13), 41.9 (C-14), 27.9 (C-15), 23.1 (C-16), 46.8 (C-17), 41.4 (C-18), 46.0 (C-19), 30.5 (C-20), 33.8 (C-21), 32.3 (C-22), 28.0 (C-23), 16.9 (C-24), 15.4 (C-25), 17.2 (C-26), 25.9 (C-27), 176.5 (C-28), 32.9 (C-29), 23.5 (C-30); ^1^H-NMR and ^13^C-NMR of sugar moieties, see Table 1.

*Oleanolic acid 3-O-β-D-glucopyranosyl(1→3)-α-L-rhamnopyranosyl(1→2)[α-L-rhamnopyranosyl(1→6)-β-D-glucopyranosyl(1→4)]-α-L-arabinopyranosyl-28-O-α-L-rhamnopyranosyl(1→4)-β-D-glucopyranosyl(1→6)-β-D-glucopyranosyl ester* (**2**). White amorphous powder; HR-TOF-MS m/z 1673.7813 [M−H]^−^ (calc. for C_77_H_126_O_39_, *m/z* 1673.7803); ^1^H-NMR of aglycone *δ*: 0.89 (1H, m, H-1a), 1.45 (1H, m, H-1b), 1.83 (1H, m, H-2a), 2.05 (1H, m, H-2b), 3.24 (1H, d, *J* = 12.0 Hz, H-3), 0.74 (1H, m, H-5), 1.60 (2H, m, H-6), 1.25 (1H, m, H-7a), 1.40 (1H, m, H-7b), 1.58 (1H, m, H-9), 0.88 (2H, m, H-11), 5.34 (1H, s, H-12), 1.09 (1H, m, H-15a), 2.27 (1H, m, H-15b), 1.91 (1H, m, H-16a), 2.02 (1H, m, H-16b), 3.12 (1H, d, *J* = 18.0 Hz, H-18), 1.19 (1H, m, H-19a), 1.69 (1H, m, H-19b), 1.06 (1H, m, H-21a), 1.29 (1H, m, H-21b), 1.69 (1H, m, H-22a), 1.90 (1H, m, H-22b), 1.27 (3H, s, H-23), 1.13 (3H, s, H-24), 0.84 (3H, s, H-25), 1.03 (3H, s, H-26), 1.20 (3H, s, H-27), 0.84 (3H, s, H-29), 0.83 (3H, s, H-30); ^13^C-NMR of aglycone *δ*: 38.8 (C-1), 26.5 (C-2), 88.5 (C-3), 39.5 (C-4), 55.9 (C-5), 18.4 (C-6), 33.0 (C-7), 39.8 (C-8), 47.9 (C-9), 36.9 (C-10), 23.7 (C-11), 122.6 (C-12), 144.9 (C-13), 42.0 (C-14), 28.1 (C-15), 23.1 (C-16), 46.9 (C-17), 41.6 (C-18), 46.1 (C-19), 30.7 (C-20), 33.9 (C-21), 32.4 (C-22), 28.2 (C-23), 17.1 (C-24), 15.6 (C-25), 17.4 (C-26), 26.0 (C-27), 176.5 (C-28), 33.0 (C-29), 23.6 (C-30); ^1^H-NMR and ^13^C-NMR of sugar moieties, see Table 2.

*Hederagenin 3-O-β-D-glucopyranosyl(1→3)-α-L-rhamnopyranosyl(1→2)[α-L-rhamnopyranosyl(1→2)-β-D-glucopyranosyl(1→4)]-α-L-arabinopyranosyl-28-O-α-L-rhamnopyranosyl(1→4)-β-D-glucopyranosyl(1→6)-β-D-glucopyranosyl ester* (**3**). White amorphous powder; HR-TOF-MS *m/z* 1689.7736 [M−H]^−^ (calc. for C_77_H_126_O_40_, *m/z* 1689.7752); ^1^H-NMR of aglycone *δ*: 1.02 (1H, m, H-1a), 1.50 (1H, m, H-1b), 1.95 (1H, m, H-2a), 2.19 (1H, m, H-2b), 4.21 (1H, m, H-3), 1.69 (1H, m, H-5), 1.60 (2H, m, H-6), 1.20 (1H, m, H-7a), 1.52 (1H, m, H-7b), 1.71 (1H, m, H-9), 1.90 (2H, m, H-11), 5.35 (1H, s, H-12), 1.15 (1H, m, H-15a), 1.65 (1H, m, H-15b), 1.95 (1H, m, H-16a), 1.99 (1H, m, H-16b), 3.12 (1H, dd, *J =* 3.6, 13.0 Hz, H-18), 1.16 (1H, m, H-19a), 1.66 (1H, m, H-19b), 1.05 (1H, m, H-21a), 1.22 (1H, m, H-21b), 1.70 (1H, m, H-22a), 1.80 (1H, m, H-22b), 3.89 (1H, m, H-23a), 4.23 (1H, m, H-23b), 1.10 (3H, s, H-24), 0.94 (3H, s, H-25), 1.06 (3H, s, H-26), 1.14 (3H, s, H-27), 0.82 (3H, s, H-29), 0.84 (3H, s, H-30); ^13^C-NMR of aglycone *δ*: 39.0 (C-1), 26.5 (C-2), 81.1 (C-3), 43.6 (C-4), 47.6 (C-5), 18.1 (C-6), 32.7 (C-7), 39.9 (C-8), 48.2 (C-9), 36.9 (C-10), 23.9 (C-11), 122.5 (C-12), 144.8 (C-13), 42.1 (C-14), 28.3 (C-15), 23.3 (C-16), 47.0 (C-17), 41.7 (C-18), 46.2 (C-19), 30.8 (C-20), 34.0 (C-21), 32.5 (C-22), 64.0 (C-23), 14.3 (C-24), 16.2 (C-25), 17.5 (C-26), 26.1 (C-27), 176.5 (C-28), 33.1 (C-29), 23.7 (C-30); ^1^H-NMR and ^13^C-NMR of sugar moieties, see Table 2.

*Gypsogenin 3-O-β-D-glucopyranosyl(1→3)-α-L-rhamnopyranosyl(1→2) [β-D-glucopyranosyl(1→4)]-α-L-arabinopyranosyl-28-O-α-L-rhamnopyranosyl(1→4)-β-D-glucopyranosyl(1→6)-β-D-glucopyranosyl ester* (**4**). White amorphous powder; HR-TOF-MS *m/z* 1541.6973 [M−H]^−^ (calc. for C_71_H_114_O_36_, *m/z* 1541.7017); ^1^H-NMR of aglycone *δ*: 1.27 (1H, m, H-1a), 1.93 (1H, m, H-1b), 1.95 (1H, m, H-2a), 2.17 (1H, m, H-2b), 4.70 (1H, m, H-3), 1.59 (1H, m, H-5), 1.12 (1H, m, H-6a), 1.75 (1H, m, H-6b), 1.49 (1H, m, H-7a), 1.75 (1H, m, H-7b), 1.93 (1H, m, H-9), 2.17 (2H, m, H-11), 5.30 (1H, s, H-12), 1.22 (1H, m, H-15a), 2.01 (1H, m, H-15b), 2.02 (1H, m, H-16a), 2.20 (1H, m, H-16b), 3.08 (1H, dd, *J =* 6.0, 12.0 Hz, H-18), 1.29 (1H, m, H-19a), 1.79 (1H, m, H-19b), 1.41 (1H, m, H-21a), 1.60 (1H, m, H-21b), 1.82 (1H, m, H-22a), 1.89 (1H, m, H-22b), 9.67 (1H, s, H-23), 1.39 (3H, s, H-24), 0.81 (3H, s, H-25), 0.95 (3H, s, H-26), 1.13 (3H, s, H-27), 0.79 (3H, s, H-29), 0.81 (3H, s, H-30); ^13^C-NMR of aglycone *δ*: 38.5 (C-1), 25.7 (C-2), 83.8 (C-3), 55.8 (C-4), 48.1 (C-5), 20.8 (C-6), 32.7 (C-7), 40.3 (C-8), 48.5 (C-9), 36.3 (C-10), 23.9 (C-11), 122.6 (C-12), 144.8 (C-13), 42.3 (C-14), 28.4 (C-15), 23.5 (C-16), 47.2 (C-17), 41.8 (C-18), 46.4 (C-19), 30.9 (C-20), 34.1 (C-21), 32.6 (C-22), 205.5 (C-23), 11.0 (C-24), 15.8 (C-25), 17.6 (C-26), 26.3 (C-27), 176.6 (C-28), 33.3 (C-29), 23.9 (C-30); ^1^H-NMR and ^13^C-NMR of sugar moieties, see Table 1.

### 3.5. Acid Hydrolysis and GC Analysis

Compound **1** (4 mg) was treated with 1 M HCl (4 mL) at 90 °C for 2 h. The reaction mixture was then extracted with CHCl_3_ (3 × 5 mL). Acid hydrolysis of **2–4** was performed likewise. Each remaining aqueous layer was concentrated to dryness to give a residue and was dissolved in pyridine (2 mL), and then L-cysteine methyl ester hydrochloride (2 mg) was added to the solution. Then the mixture was heated at 60 °C for 1 h, and trimethylchlorosilane (0.5 mL) was added, followed by heating at 60 °C for 30 min. Then, the solution was concentrated to dryness and taken up in water (1 mL × 3), followed by extraction with n-hexane (1 mL × 3). The supernatant was subjected to GC analysis under the following conditions: Agilent GC 6890 instrument equipped with FID (detection temperature 280 °C). Column: HP-5 column (320 μm × 30 m, 0.25 μm). Column temperature: 160–200 °C with the rate of 4 °C/min, then kept for 5 min, and then 200–240 °C with the rate of 10 °C/min and kept for 10 min. The carrier gas was N_2_ (1.0 mL/min), split ratio 1/10, injection temperature: 270 °C. Injection volume: 10 μL. The absolute configurations of the monosaccharides were confirmed to be L-arabinose, L-rhamnose, and D-glucose by comparison of the retention times of monosaccharide derivatives with those of standard samples: L-arabinose (12.67 min), L-rhamnose (12.85 min), and D-glucose (14.41 min), respectively.

### 3.6. Cell Culture

Human neuroblastoma SH-SY5Y cells were cultured using DMEM/F12 culture (Gibco, USA), supplemented with 10% (v/v) fetal bovine serum (Therom Scientific, USA), 100 U/mL penicillin and 100 μg/mL streptomycin at pH 7.4 and maintained in a humidiﬁed 5% CO_2_ atmosphere at 37 °C. Experiment was carried out 24 h after cells were seeded.

### 3.7. Determination of Cell Viability

Cell viability was assessed using conventional 3-[4,5-dimethylthiazolo-2]-2,5-diphenyltetrazolium bromide (MTT) assay. Aβ_25–35_ (Sigma-Aldrich, USA) was dissolved in distilled water and aged at 37 °C for 7 days before use. The cultured cells were pre-incubated with different compounds for 18 h in 96-well plates, and exposed to 20 μM Aβ_25–35_ for an additional 30 h, then 15 μL of MTT stock solution (5 mg/mL) (Sigma-Aldrich, USA) was added to the culture medium for 4 h at 37 °C. The MTT formazan crystals were solubilized by 150 μL DMSO and the absorbance was measured at 492 nm using a microplate reader (Tecan, Switzerland). Results were expressed as percentage of control.

### 3.8. Statistical Analysis

The results of cell viability were expressed as mean ± S.E.M. One-way ANOVA followed by Dunnett’s t-test was performed to statistical analysis. *p* < 0.05 was considered statistically significant.

## 4. Conclusions

Four new oleanene-type triterpenoid saponins together with six known saponins were isolated from the roots of *P. cernua* and their structures were elucidated on the basis of spectroscopic data, including 2D NMR spectra and chemical evidence. Among these compounds **9** was isolated for the first time from this genus, and **5**, **6**, and **10** were reported from this plant for the first time. Furthermore, the aglycone of **4** has not been reported from this genus before. Interestingly, other aglycones with aldehyde groups at C-23 have not been obtained in this genus, either, which suggested that there might be some specific enzyme system in *P.cernua* compared with other plants of this genus. Compounds **1**, **4**, **7** and **8** showed significant effect against the cytotoxicity induced by Aβ_25–35_ on SH-SY5Y cell which suggested that these compounds might be good candidates for the prevention and treatment of AD, and can be further investigated.

## Supplementary Materials

Supplementary materials can be accessed at: http://www.mdpi.com/1420-3049/17/5/5520/s1.
