# Supplementary material for: Saponins with Neuroprotective Effects from the Roots of Pulsatilla cernua
Source: Molecules. 2012 May 9;17(5):5520–31. doi: 10.3390/molecules17055520 (PMC6268475; doi:10.3390/molecules17055520)

Saponins with Neuroprotective Effects from the Roots of *Pulsatilla cernua*

Supplementary Materials

S1. ^{1}H-NMR spectrum (600 MHz, pyridine-d_5) of compound **1**.
S2. ^{13}C-NMR spectrum (150 MHz, pyridine-d_5) of compound **1**
S3. HSQC spetrum of compound **1**.
S4. HMBC spetrum of compound **1**.
S5. HSQC-TOCSY spetrum of compound **1**.
S6. ^{1}H-NMR spectrum (600 MHz, pyridine-d_5) of compound **2**.
S7. ^{13}C-NMR spectrum (150 MHz, pyridine-d_5) of compound **2**
S8. HSQC spetrum of compound **2**.
S9. HMBC spetrum of compound **2**.
S10. ^{1}H-NMR spectrum (600 MHz, pyridine-d_5) of compound **3**.
S11. ^{13}C-NMR spectrum (150 MHz, pyridine-d_5) of compound **3**
S12. HSQC spetrum of compound **3**.
S13. HMBC spetrum of compound **3**.
S14. ^{1}H-NMR spectrum (600 MHz, pyridine-d_5) of compound **4**.
S15. ^{13}C-NMR spectrum (150 MHz, pyridine-d_5) of compound **4**

S1. ^{1}H-NMR spectrum (600 MHz, pyridine-d_5) of compound **1**.

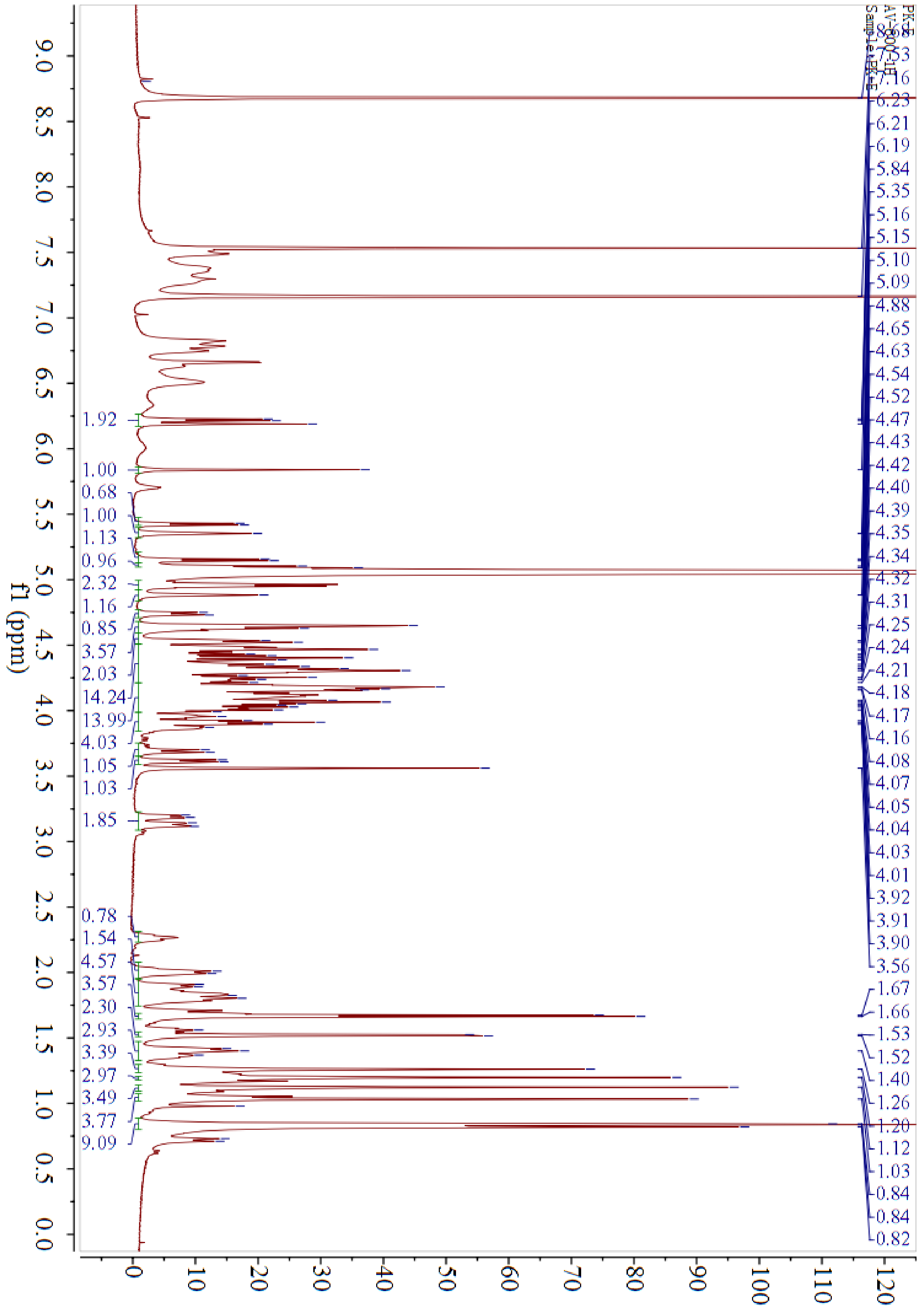

S2. ^{13}C-NMR spectrum (150 MHz, pyridine-d_5) of compound **1**.

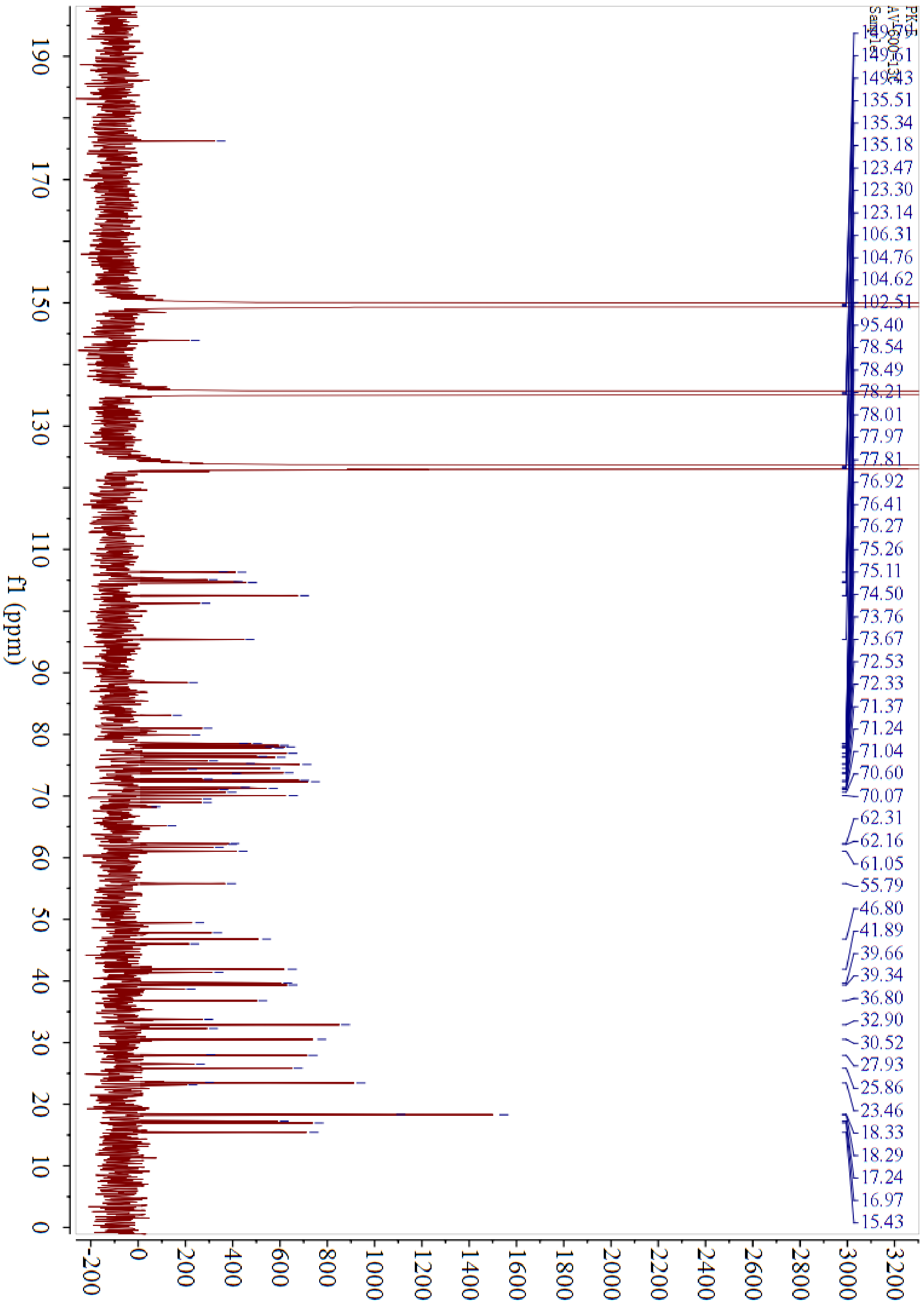

S3. HSQC spetrum of compound **1**.

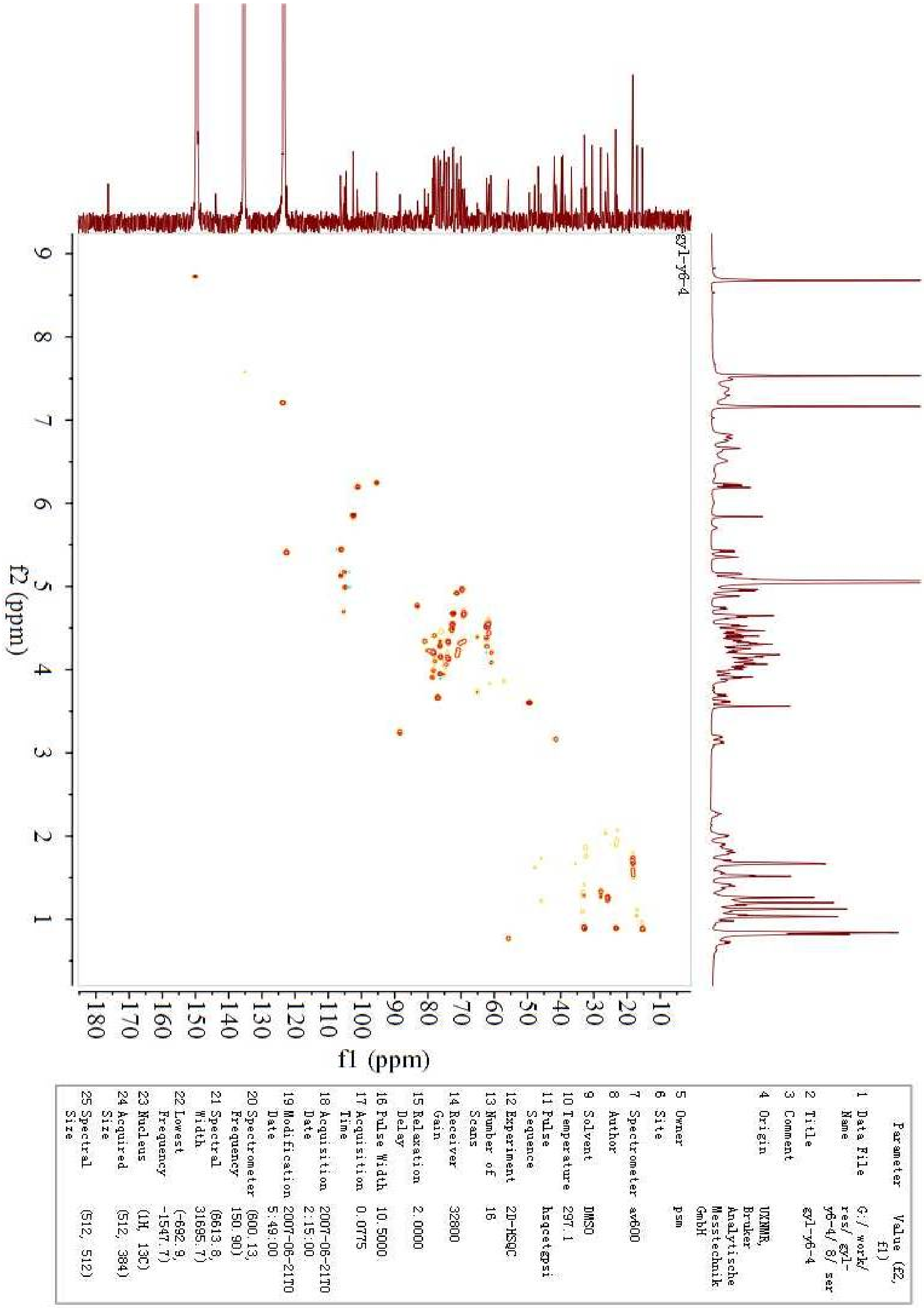

S4. HMBC spetrum of compound **1**.

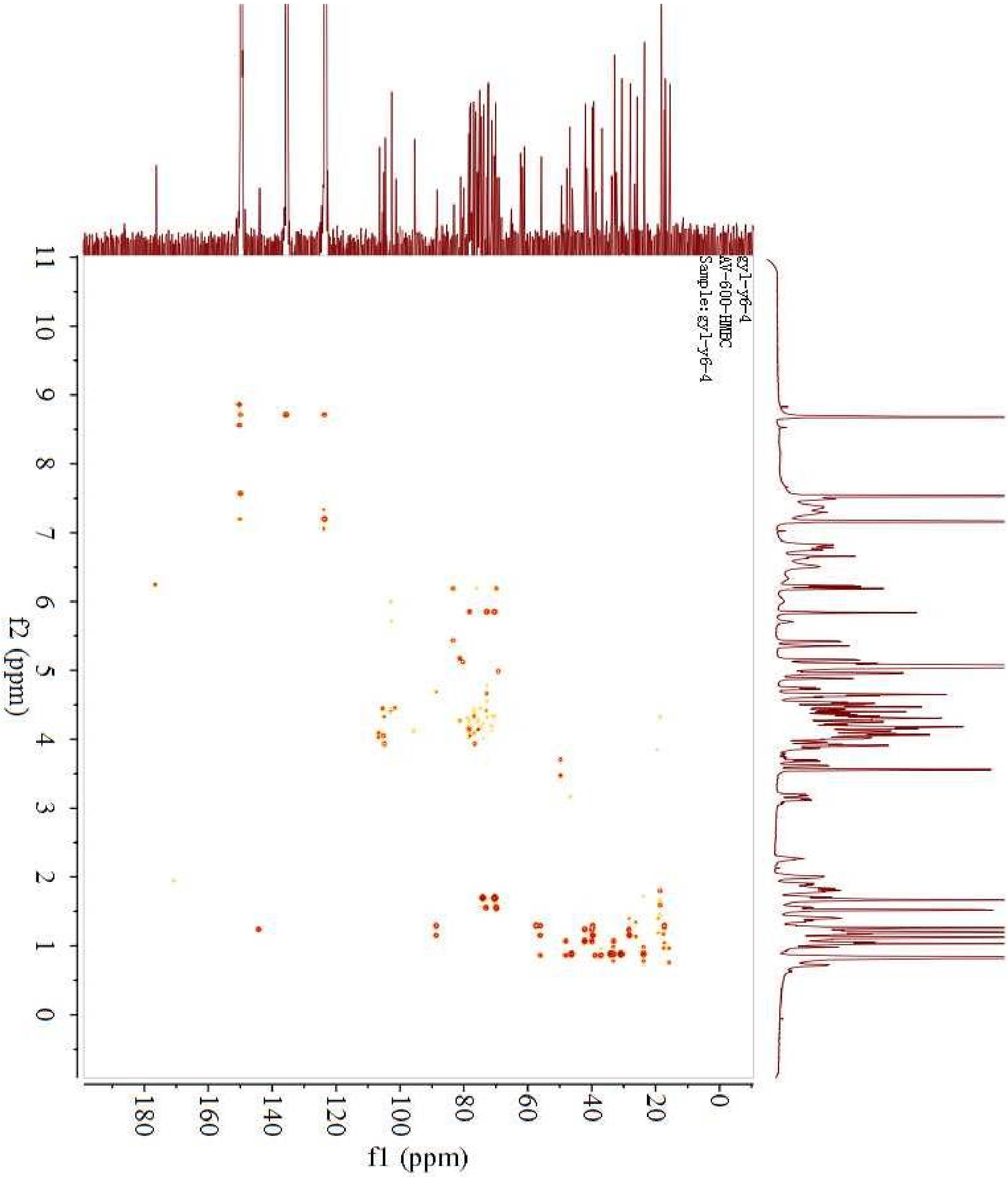

Parameter	Value (f2, f1)
1 Data File Name	G:/ work/ res/ gyl-y6-4/ 9/ ser
2 Title	gyl-y6-4
3 Comment	AV-600-HMBC Sample:gyl-y6-4
4 Origin	UXNMR, Bruker Analytische Messtechnik GmbH
5 Owner	root
6 Site	
7 Spectrometer	av600
8 Author	
9 Solvent	Pyr
10 Temperature	297.3
11 Pulse Sequence	hmbcgplpndqf
12 Experiment	2D-HMBC
13 Number of Scans	64
14 Receiver Gain	29100
15 Relaxation Delay	2.0000
16 Pulse Width	10.5000
17 Acquisition Time	0.0714
18 Acquisition Date	2007-06-03T09:59:00
19 Modification Date	2007-06-03T23:09:00
20 Spectrometer Frequency	(600.13, 150.90)
21 Spectral Width	(7183.9, 31695.7)
22 Lowest Frequency	(-564.5, -1674.0)
23 Nucleus	(1H, 13C)
24 Acquired Size	(512, 340)
25 Spectral Size	(512, 512)

S5. HSQC-TOCSY spetrum of compound **1**.

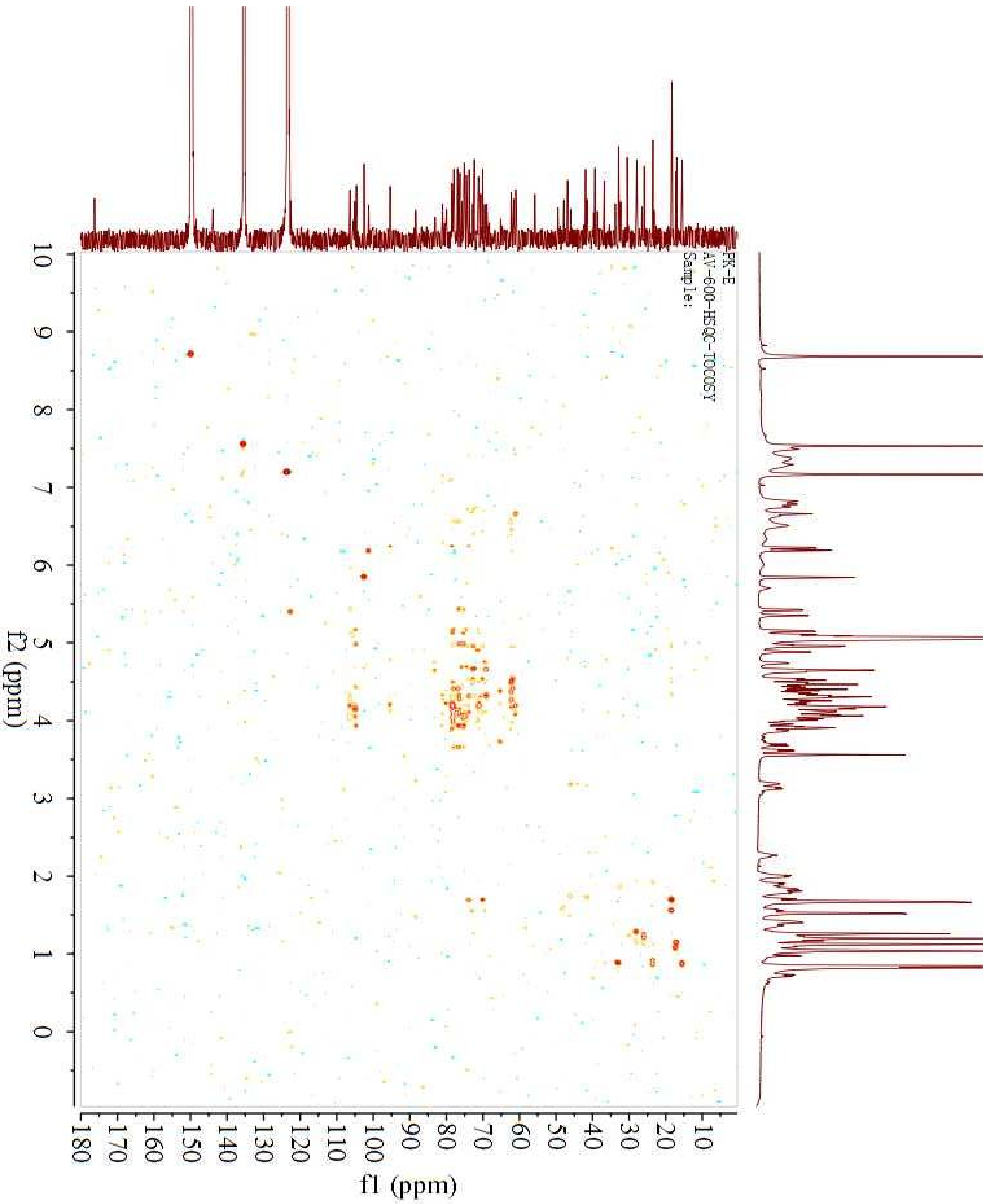

	Parameter	Value (f2, f1)
1	Data File Name	G:/ work/ res/ PK-E/ 11/ ser
2	Title	PK-E
3	Comment	AV-600-HSQC-TOCSY Sample:
4	Origin	Bruker BioSpin GmbH
5	Owner	av600
6	Site	
7	Spectrometer	spect
8	Author	
9	Solvent	Pyr
10	Temperature	298. 2
11	Pulse Sequence	hsqcgpmlph
12	Experiment	2D-HSQC-TOCSY
13	Number of Scans	16
14	Receiver Gain	18400
15	Relaxation Delay	1. 5000
16	Pulse Width	10. 5000
17	Acquisition Time	0. 0774
18	Acquisition Date	2012-04-27T18:14:59
19	Modification Date	2012-04-27T21:07:00
20	Spectrometer Frequency	(600. 13, 150. 90)
21	Spectral Width	(6613. 8, 27165. 0)
22	Lowest Frequency	(-593. 6, 8. 8)
23	Nucleus	(1H, 13C)
24	Acquired Size	(512, 384)
25	Spectral Size	(512, 512)

S6. ^{1}H-NMR spectrum (600 MHz, pyridine-d_5) of compound **2**.

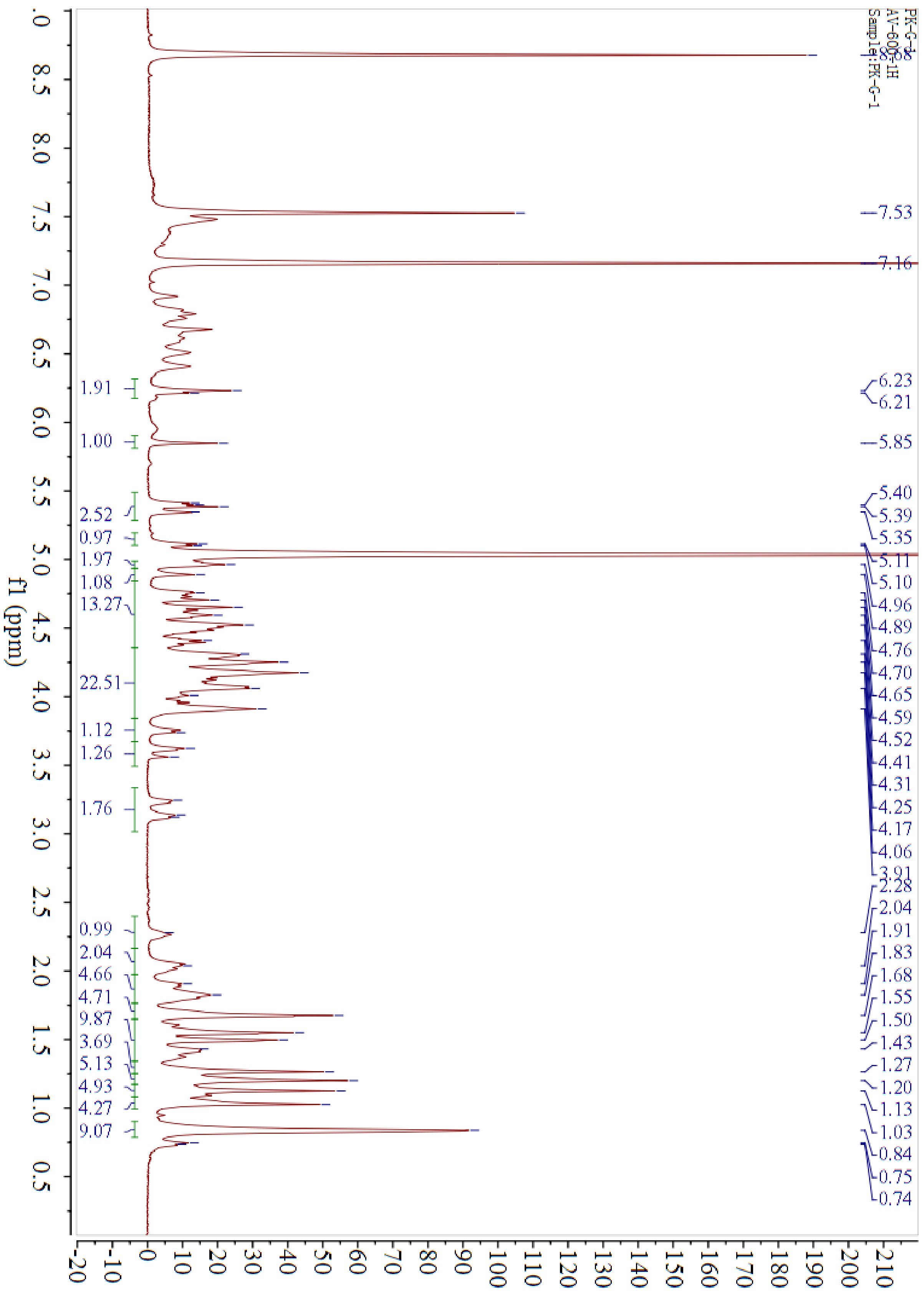

S7. ^{13}C-NMR spectrum (150 MHz, pyridine-d_5) of compound **2**

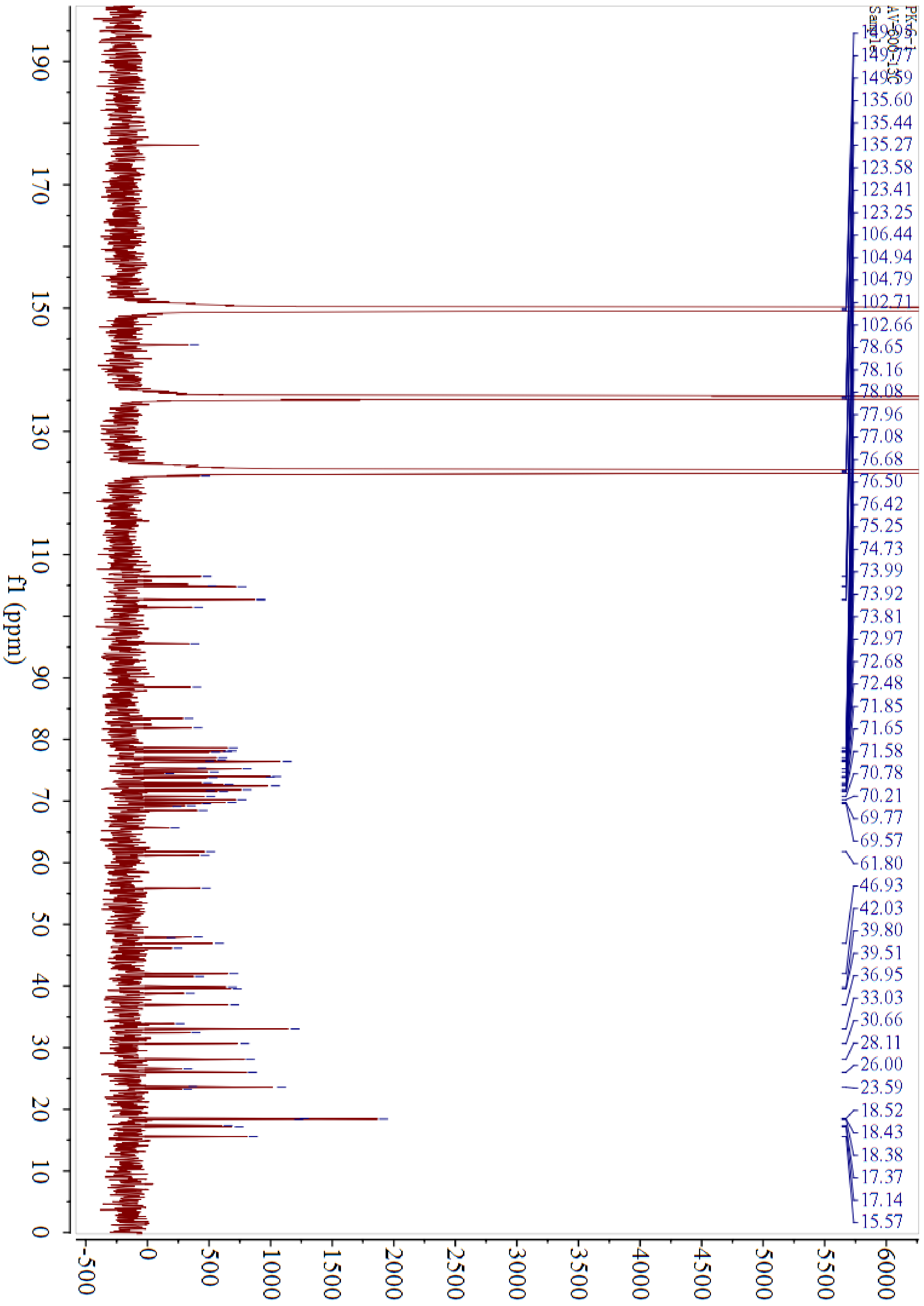

S8. HSQC spetrum of compound **2**.

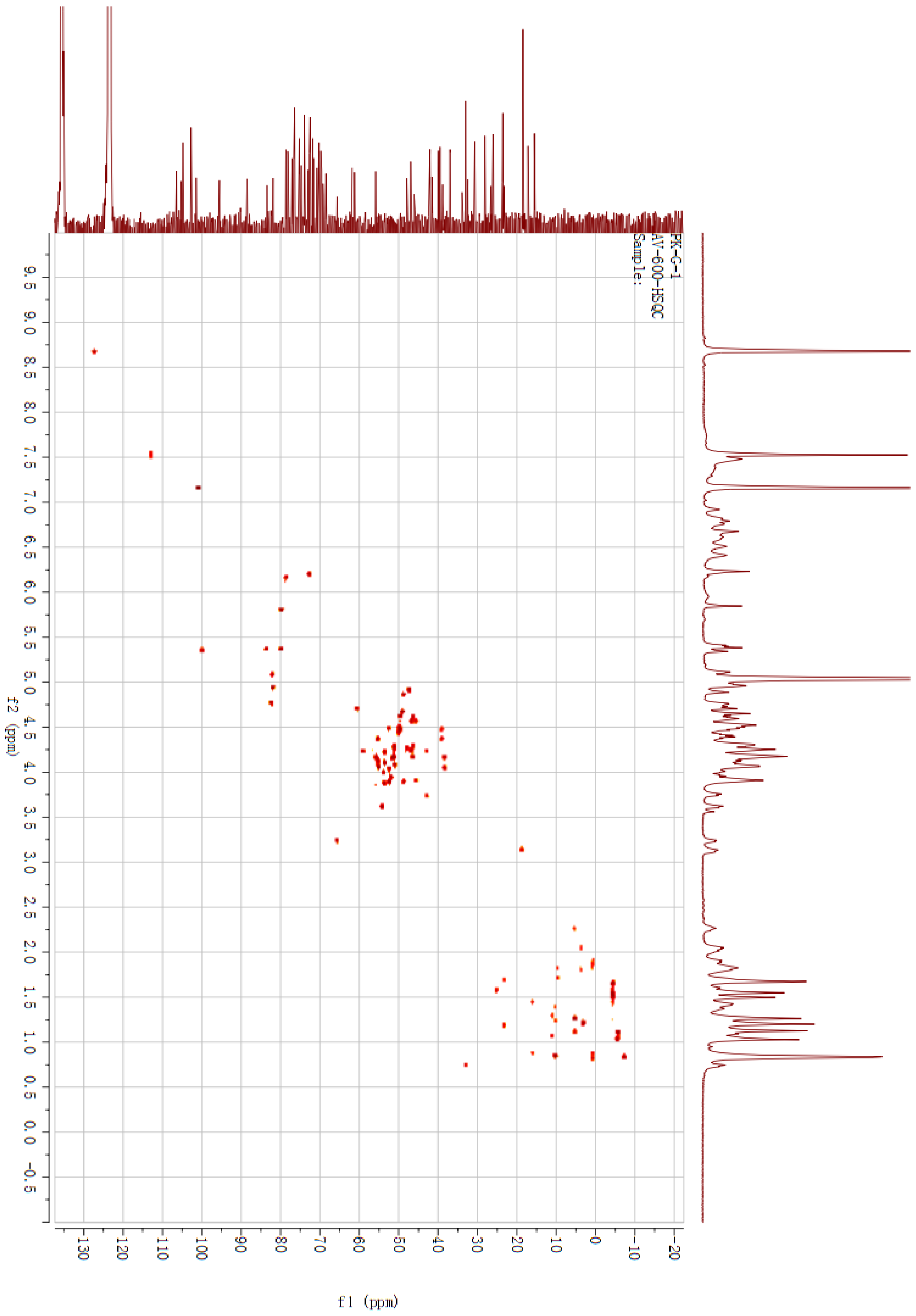

S9. HMBC spetrum of compound **2**.

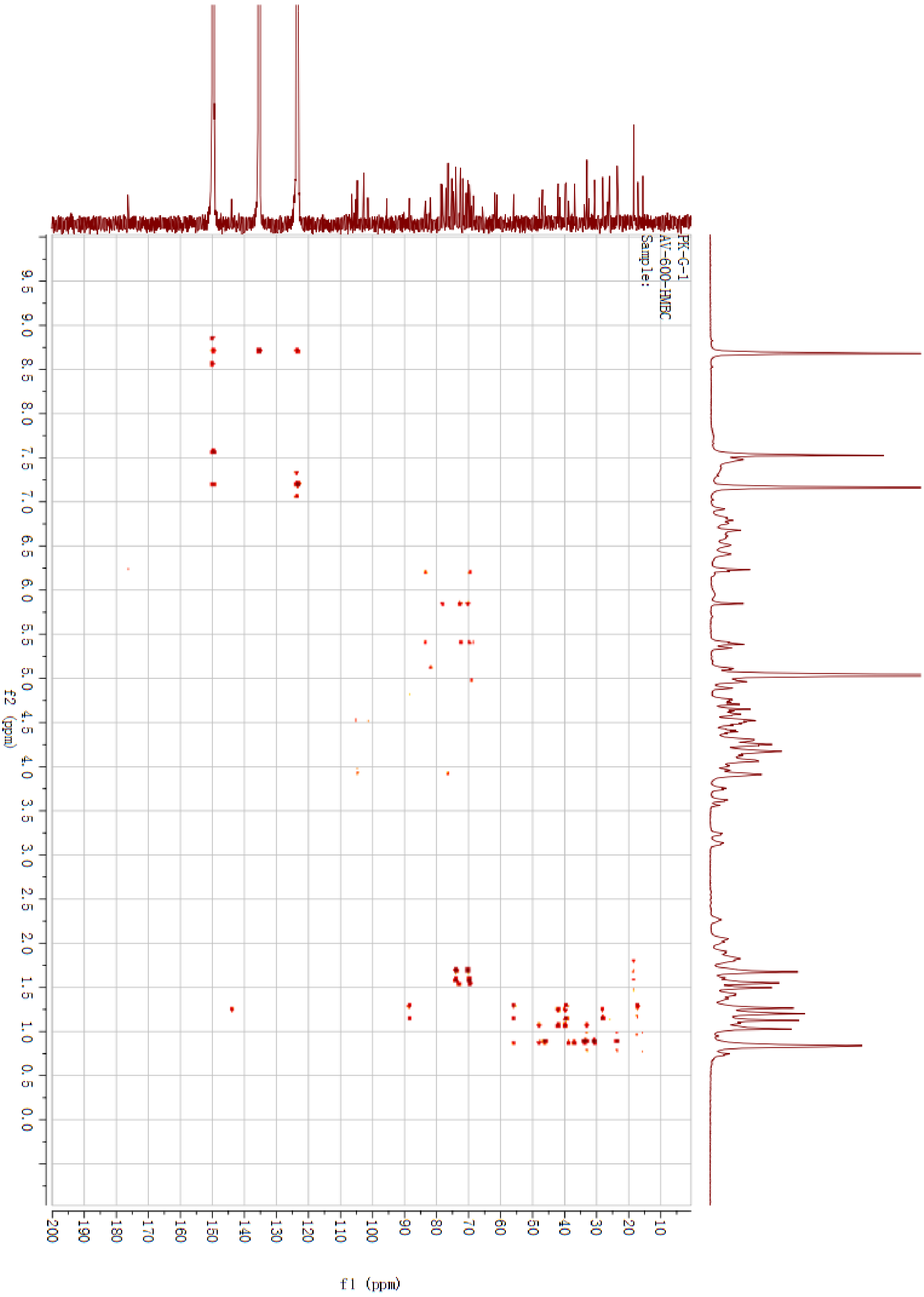

S10. ^{1}H-NMR spectrum (600 MHz, pyridine-d_5) of compound **3**.

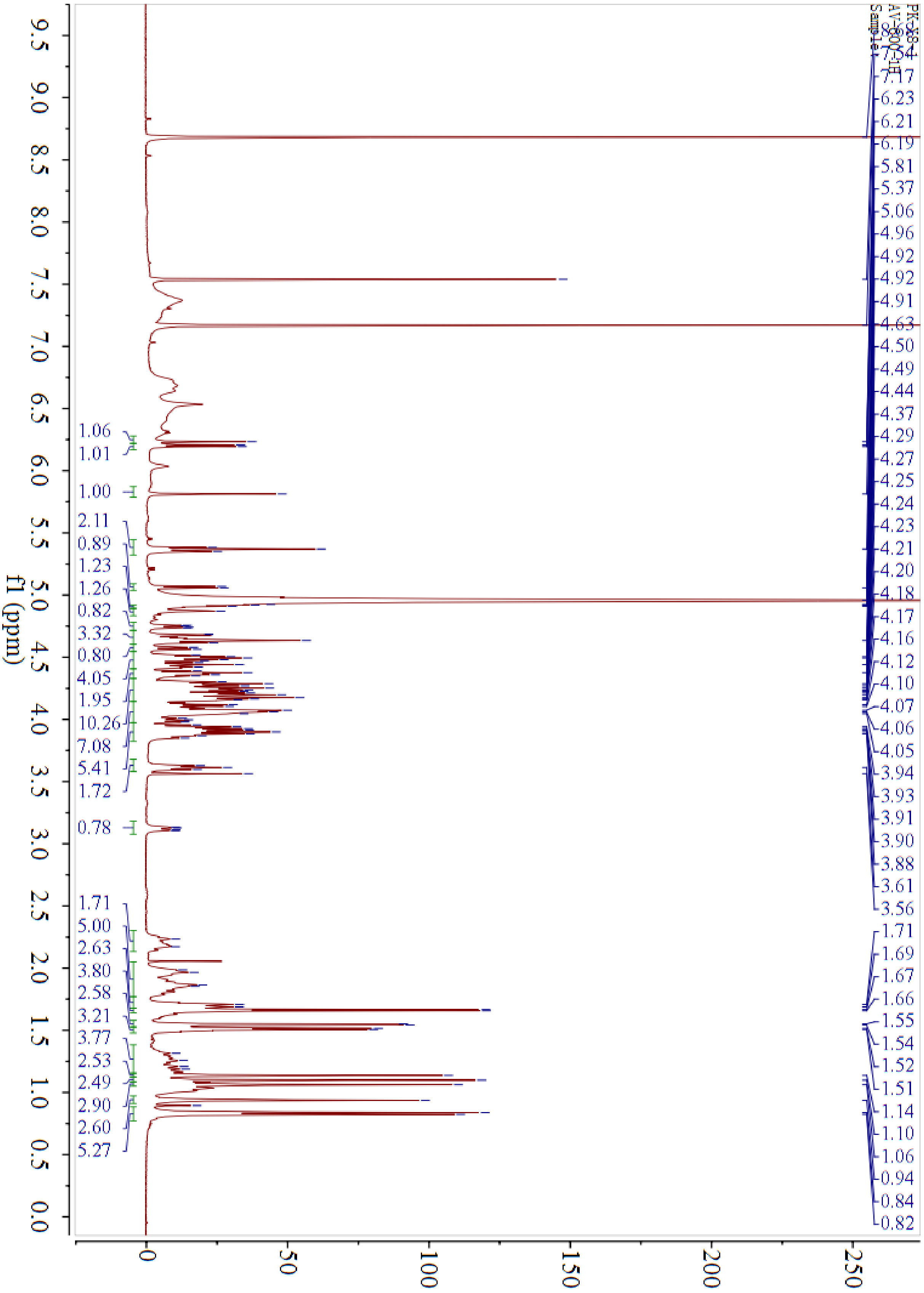

S11. ^{13}C-NMR spectrum (150 MHz, pyridine-d_5) of compound **3**.

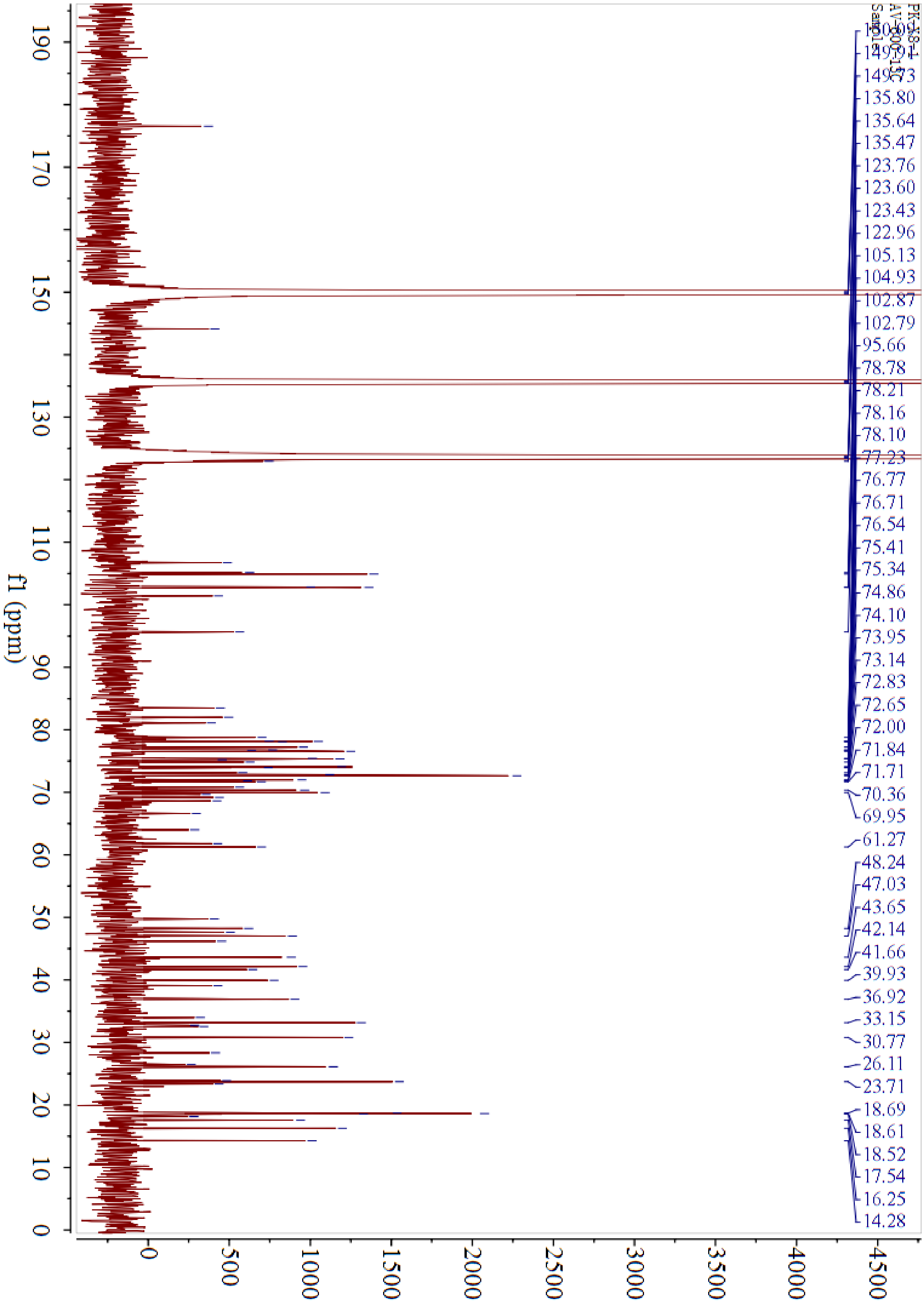

S12. HSQC spetrum of compound **3**.

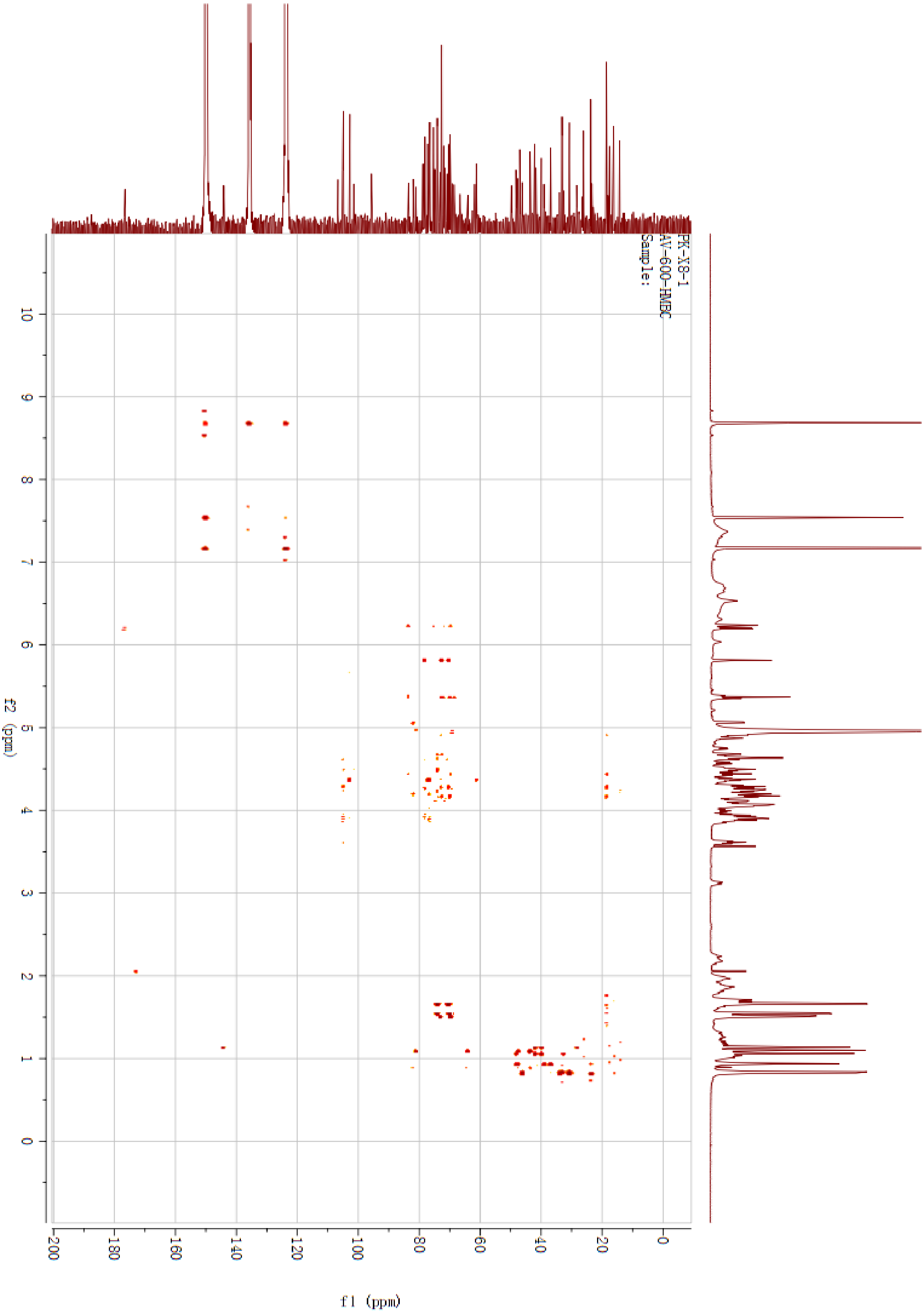

S13. HMBC spetrum of compound **3**.

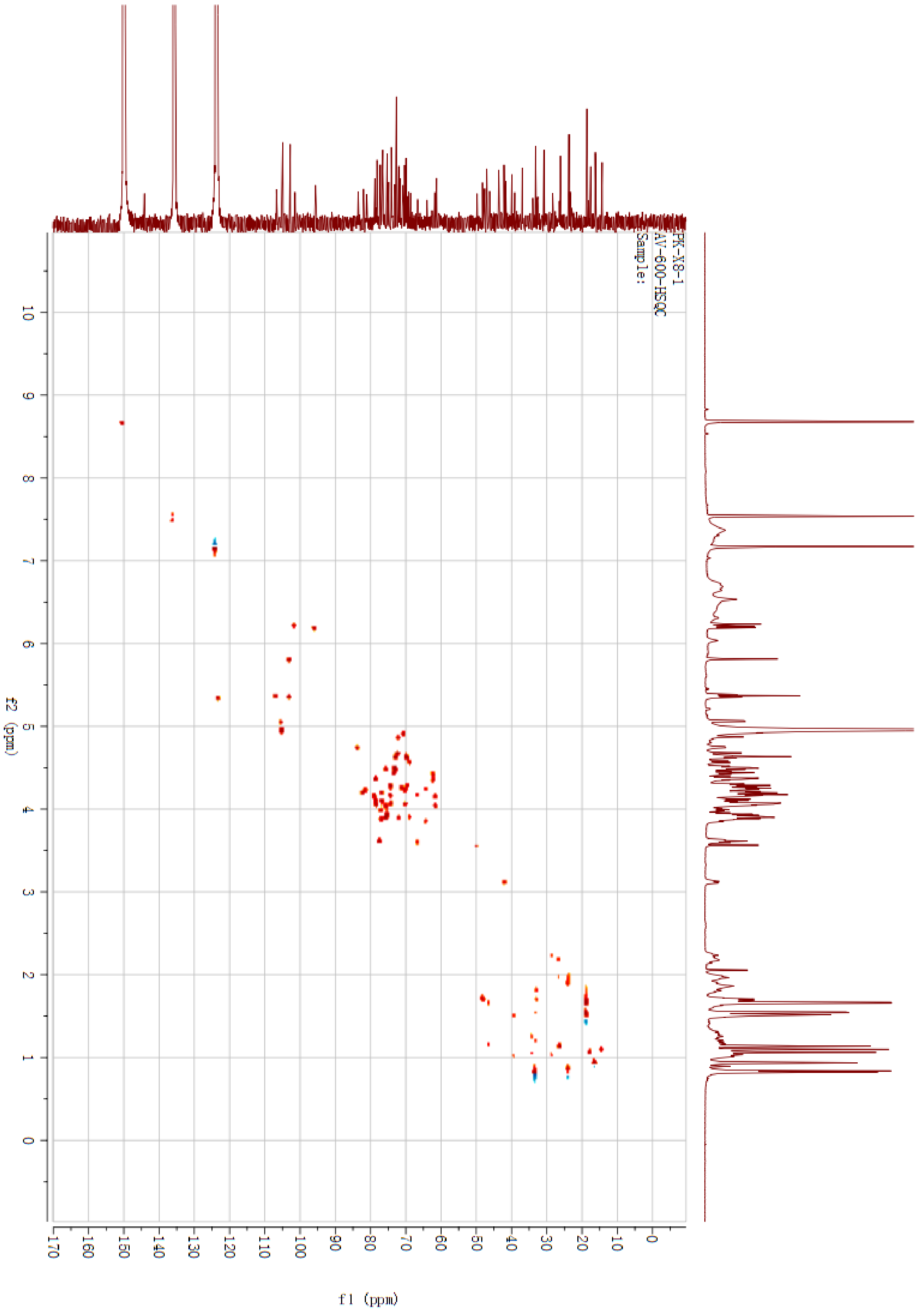

S14. ^{1}H-NMR spectrum (600 MHz, pyridine-d_5) of compound **4**.

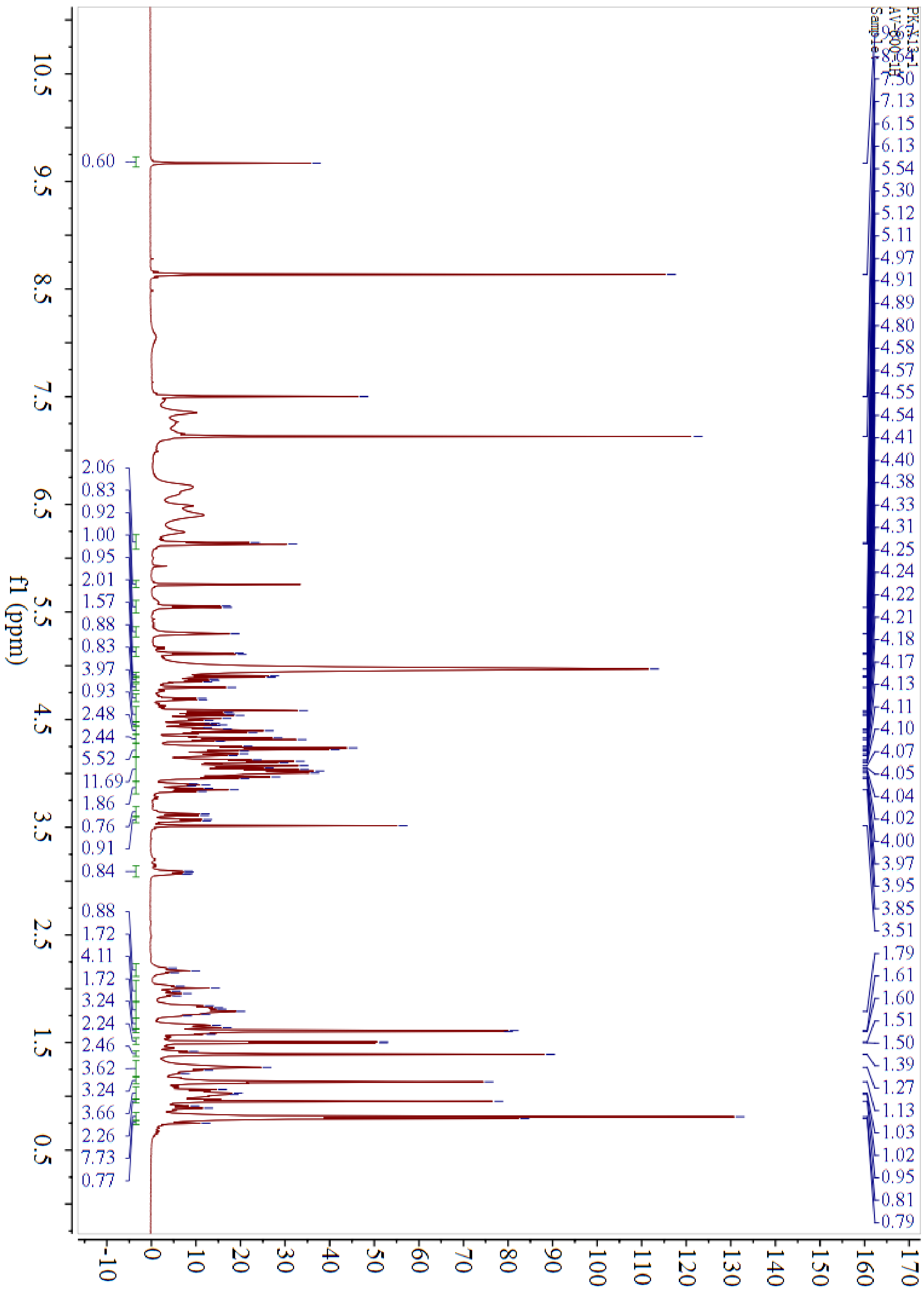

S15. ^{13}C-NMR spectrum (150 MHz, pyridine-d_5) of compound **4**.

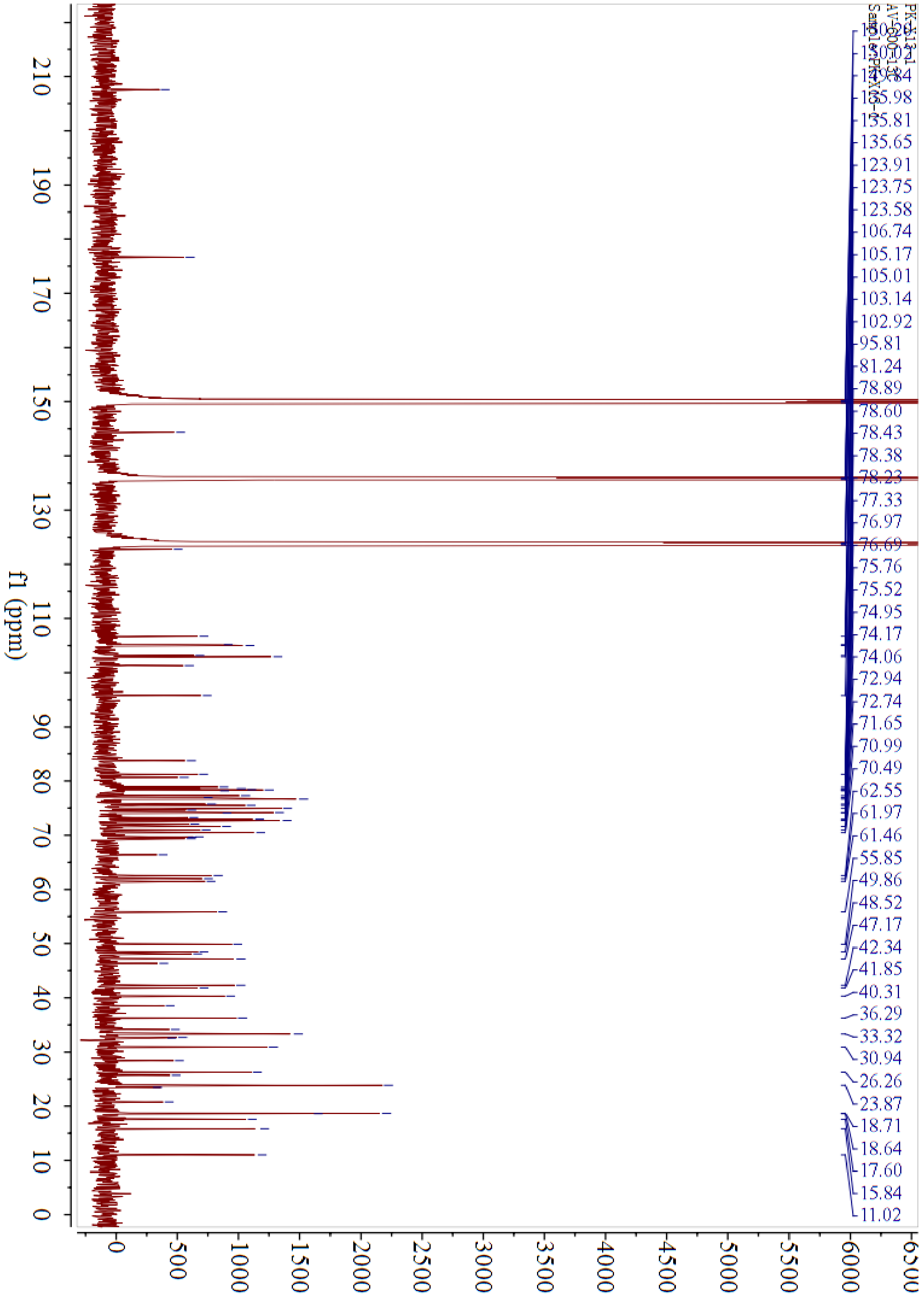

Supplement: Supplementary file 1 [file molecules-17-05520-s001.pdf]
